# Supplementary figures and images for: Effect of Salinity on Stomatal Conductance, Leaf Hydraulic Conductance, HvPIP2 Aquaporin, and Abscisic Acid Abundance in Barley Leaf Cells
Source: Int J Mol Sci. 2022 Nov 18;23(22):14282. doi: 10.3390/ijms232214282 (PMC9694007; doi:10.3390/ijms232214282)

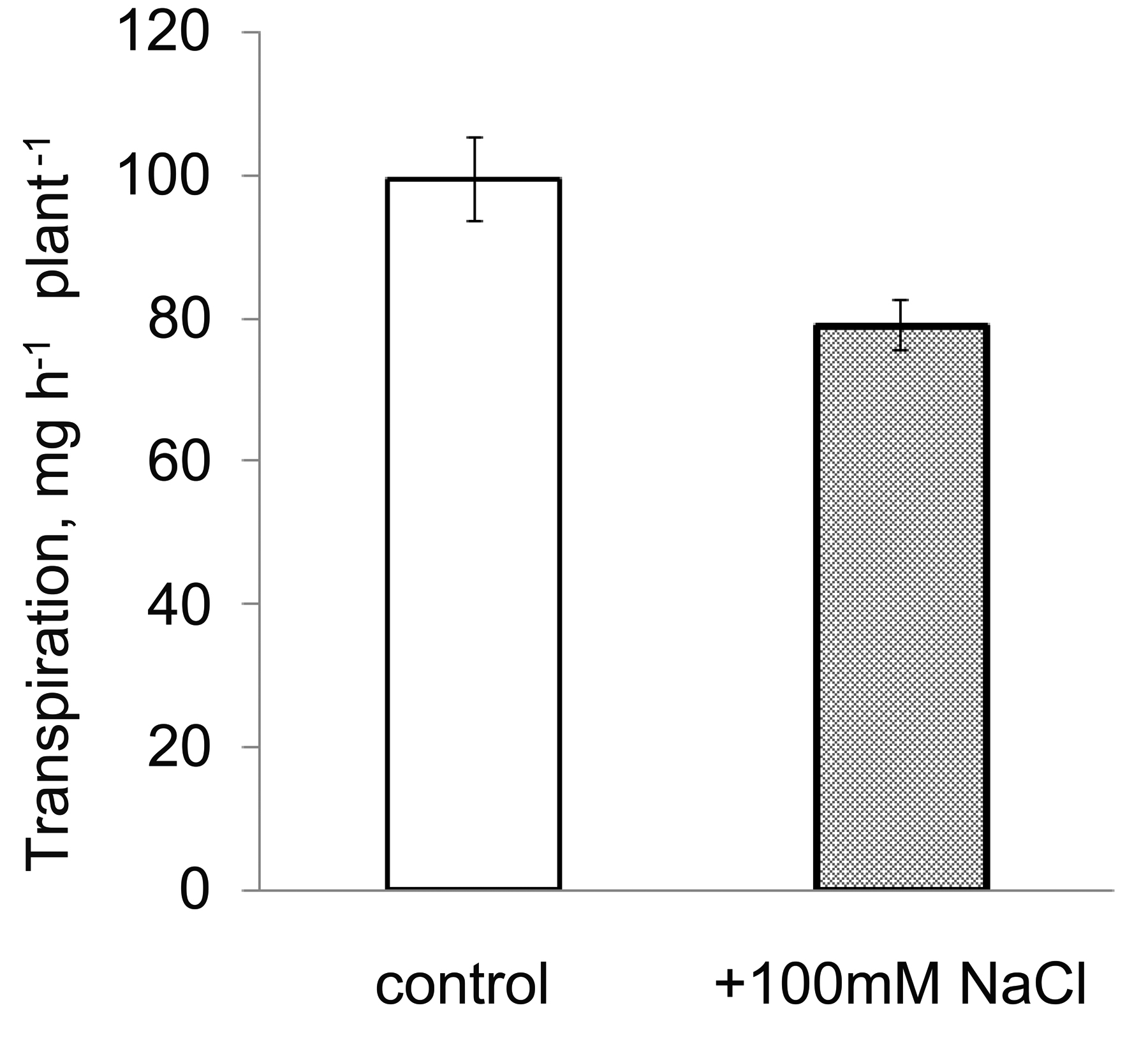

Supplement: Supplementary file 1 [file ijms-23-14282-s001.zip › ijms-1992450-supplementary.jpg]
